# Visualizing Molecular Profiles of Glioblastoma with GBM-BioDP

**DOI:** 10.1371/journal.pone.0101239

**Published:** 2014-07-10

**Authors:** Orieta Celiku, Seth Johnson, Shuping Zhao, Kevin Camphausen, Uma Shankavaram

**Affiliations:** Radiation Oncology Branch, National Cancer Institute, National Institutes of Health, Bethesda, Maryland, United States of America; Beijing Tiantan Hospital, Capital Medical University, China

## Abstract

Validation of clinical biomarkers and response to therapy is a challenging topic in cancer research. An important source of information for virtual validation is the datasets generated from multi-center cancer research projects such as The Cancer Genome Atlas project (TCGA). These data enable investigation of genetic and epigenetic changes responsible for cancer onset and progression, response to cancer therapies, and discovery of the molecular profiles of various cancers. However, these analyses often require bulk download of data and substantial bioinformatics expertise, which can be intimidating for investigators. Here, we report on the development of a new resource available to scientists: a data base called Glioblastoma Bio Discovery Portal (GBM-BioDP). GBM-BioDP is a free web-accessible resource that hosts a subset of the glioblastoma TCGA data and enables an intuitive query and interactive display of the resultant data. This resource provides visualization tools for the exploration of gene, miRNA, and protein expression, differential expression within the subtypes of GBM, and potential associations with clinical outcome, which are useful for virtual biological validation. The tool may also enable generation of hypotheses on how therapies impact GBM molecular profiles, which can help in personalization of treatment for optimal outcome. The resource can be accessed freely at http://gbm-biodp.nci.nih.gov (a tutorial is included).

## Introduction

The Cancer Genome Atlas (TCGA) project [1] has created a wealth of multi-center, multi-dimensional genomic data on more than 20 cancers. Studies based on these data have improved our understanding of molecular profiles of different cancers, regulatory networks that change during disease, and molecular markers and key targets for therapy.

The expanding quantity of data and proliferation of results from computational analyses create the challenge of making the experimental data and accumulated knowledge accessible to a wide range of researchers. Bulk downloads and re-processing of data is unappealing in most situations and only an option to bioinformatics experts in search of new ways to analyze the data. Most researchers and bench scientists need easily-accessible data browsers and visualization tools that present abstracted, intuitive, and integrated views of the data, and that enable validation of biological insights, or generation of new hypotheses. To this end we present the Glioblastoma BioDiscovery Portal (GBM-BioDP) – an intuitive, integrative, web-accessible data portal that contains a subset of the TCGA glioblastoma (GBM) data, and incorporates knowledge from computational analyses and predictions.

GBM is the most common and aggressive malignant primary brain tumor. Patients with GBM have a poor prognosis and usually the standard first-line of treatment is surgery, followed by radiation therapy or combined radiation and chemotherapy (such as temozolomide). Unfortunately, these treatments are rarely curative and the vast majority of tumors recur locally within the brain, with a median survival of 15 months [2]. Although a subset of patients containing methylated O6-methylguanine-DNA methyltransferase (MGMT) promotor show a better response to treatment with a median survival of 22 months [3], there is urgent need for better second-line therapies for recurrent GBM that responds poorly to first-line therapies [4], [5]. Currently, there are no effective long term treatments for this disease. Some of the chemotherapy strategies used for GBM includes several combinations of platinum, procarbazine, enzastaurin, and carboplatin with controversial results [2]. Of these studies, a new treatment regimen reported by Addeo et al, using a biweekly induction schedule of fotemustine (FTM) in TMZ-pretreated patients which showed promise with an increased efficacy and a favorable safety profile as a single-drug second-line chemotherapy [5].

GBM is extremely heterogeneous and arises from dysregulation of a number of important biological pathways, which makes understanding the responsible molecular mechanisms difficult. Nevertheless, computational analyses of multidimensional TCGA data are improving our understanding of the disease. Four molecular subtype profiles of the disease have been identified by computational tools: *classical*, *mesenchymal*, *proneural*, and *neural* (C, M, P, N) [6]. These subtypes are morphologically indistinguishable but exhibit markedly different molecular profiles, characteristics, and even prognosis (see Olar et al. [7] for a recent review of how these differences are informing personalized treatment). The classical subtype is associated with amplification of EGFR and EGFR vIII mutations, but typically no TP53 mutations. The neural profile is closer to the normal neural molecular profile. The proneural subtype is typically associated with younger age at diagnosis, IDH1/2 and TP53 mutations, and better prognosis. The mesenchymal subtype has more frequent NF1 mutations and exhibits enrichment of gene signatures associated with an Epithelial to Mesenchymal Transition.

GBM-BioDP has two main uses: biological validation, and new hypothesis generation. First, it can answer gene or miRNA specific questions, such as: Is a gene of interest changed in any of the molecular subtypes of GBM? Is its expression level associated with the clinical outcome of GBM patients? Do the expression and target prediction data support a regulatory relationship between given genes and miRNAs? Many publications have identified specific genes or miRNAs whose dysregulation has been associated with GBM and whose expression levels are associated with better or worse prognosis. For bench scientists our resource makes it possible to explore the stipulated relationships visually. The second way the resource can be utilized is as a sand box for hypothesis generation. Cancer therapies impact the cancer molecular profiles. User provided expression data can be compared against the known GBM subtypes and classified using several prediction models. For treated conditions the user can inspect whether treatment modifies the classification, thus making the cancer more or less aggressive.

Related tools for analyzing, integrating, and visualizing cancer genomic data have been developed alongside experimental data generation. A recent overview of cancer genomic visualization tools is given by Schroeder et al. [8]. They identify three complementary approaches to visualizing high-throughput multi-omic cancer data centered on 1) genome coordinates (eg. IGV [9], Savant [10]), 2), 2) gene-based heatmaps (eg. UCSC Cancer Genomics Browser [11], [12], Gitools [13], cBioPortal [14], [15], StratomeX [16], CircleMap [17]), and 3) networks (Cytoscape [18], [19]). As far as other features are concerned visualization tools vary on whether they are proprietary or open access, stand-alone or web accessible, degree of bioinformatics expertise assumed of the user, whether the user can upload data or not, and the various degrees in which they incorporate annotations such as clinical information.

Two of the most commonly used freely available, web-accessible tools are the UCSC Cancer Genomics Browser [11], [12], and cBioPortal [14], [15]. They host and display multi cancer, multi omic data. UCSC Cancer Genomics Browser provides options for generating heatmaps of data from individual platforms, includes rich annotations of data (such as G-Cimp status, expression subtype), and enables survival analysis. For the moment, no miRNA data are included, and no integration of the different type of data is performed. cBioPortal has a strong emphasis on visualization of mutation data (including across different cancers, and at the gene set level), but also provides views for exploring the relationship of mutation data to gene expression for individual genes, and pairs of genes. cBioPortal does not include miRNA data either. Both these tools and others offer complex visualization options, however no tool fits all purposes all the time.

Some of the features that make our tool unique are: multiple ways of stratifying samples when evaluating clinical impact (such as by GBM subtype, length of survival, or multi (gene/protein/miRNA) prognostic index), aggregation of expression data from different platforms, incorporation of miRNA expression as well as regulator-target relationship predictions, and classification of user-input expression data. We are also working towards integration of other dimensions, such as copy number, and DNA methylation.

In the reminder of this article we describe the tool construction, core features, and use cases.

## Materials and Methods

### Experimental and clinical data

Experimental and clinical data were downloaded from the TCGA data portal (https://tcga-data.nci.nih.gov/) as described in The Cancer Genome Atlas research Network [20].

- Gene expression data include normalized (level 3) data from three platforms HT_HG-U133A (488 patient samples×12042 features), HuEx-1_0-st-v2 (437 patient samples×18631 features), AgilentG4502A_07_1/2 (101+396 patient sample×17813 features). The data from the three platforms were aggregated following [6]. For a given gene and sample featured in each of the platforms, an aggregated score was computed by weight averaging the individual platforms’ scores, with scores in closer agreement being given a larger weight.
- Protein expression data include normalized (level 2) Reverse Phase Protein Array data (217 patient samples×171 features). 45 of the featured antibodies interrogate the phosphorylation status of the proteins.
- miRNA expression consist of normalized (level 3) data from H-miRNA_8×15 K platform (436 patient samples×534 features).
- Clinical data include partial clinical information on 564 patients.

### Data processing

The experimental (level 2 and 3) data were already pre-processed as part of the TCGA. For protein expression (level 2), preprocessing include: centering, z-score transformation with corresponding p-values, and summarizations such as percentage of under/over-expressed features.

### Computational and meta data

Data from several types of computational analysis were incorporated in the knowledge base.

Verhaak et al. [6] classified GBM into four types – *classical*, *mesenchymal*, *proneural*, and *neural* (C, M, P, N). Data from Verhaak et al. [6] comprising gene expression and molecular subtype classification of 201 GBM patients were used for training supervised models using two methods: predictive analysis of microiarrays (PAM) [21] and Random Forest [22]. The trained models were used to predict the molecular subtype of an additional 201 of the remaining patient samples from the database.

We also incorporate computational predictions of miRNA targets from MicroCosm. Predicted targets can be used in conjunction with expression data to examine regulator-target relationships between miRNAs and genes or proteins.

### Construction

**Figure 1:**
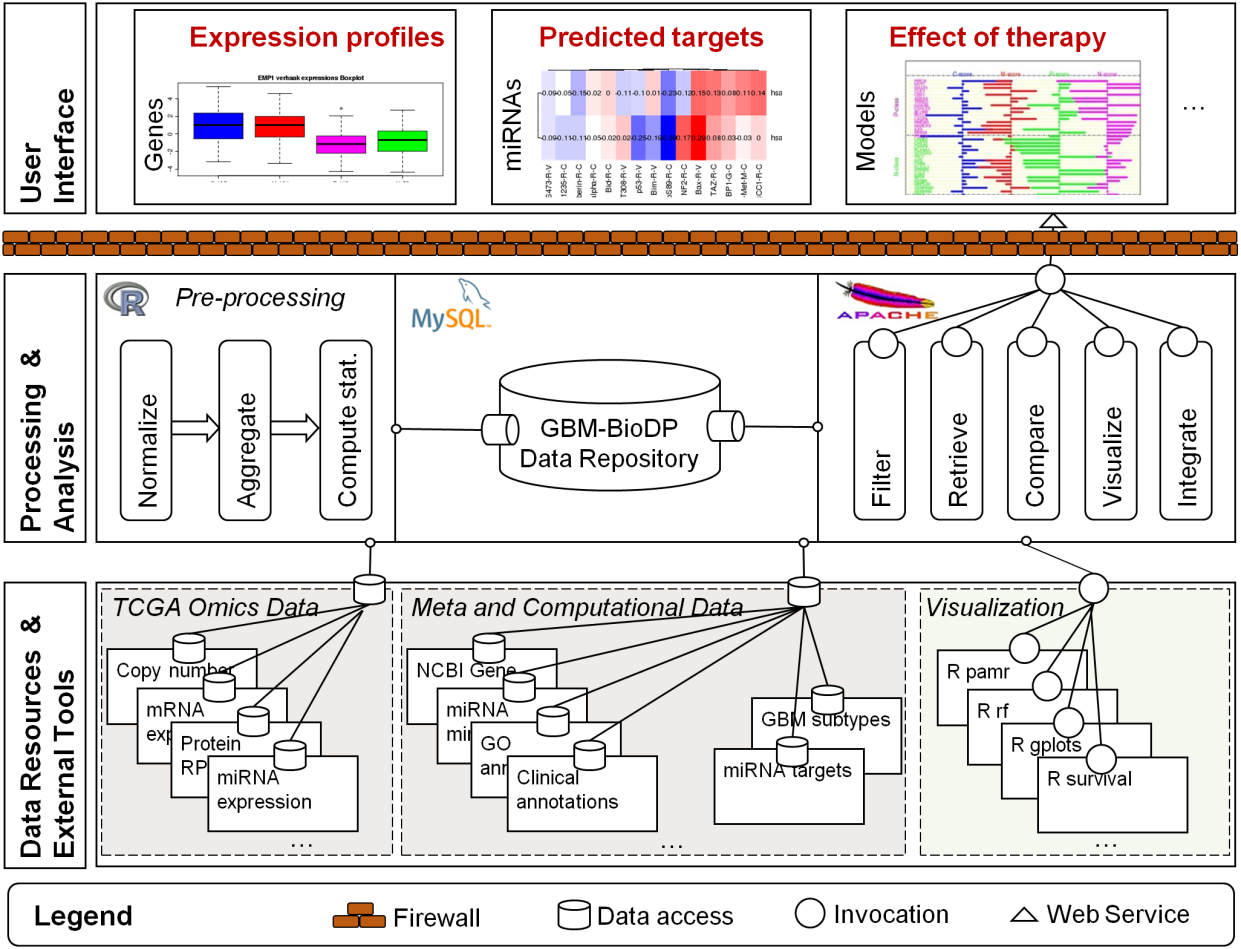
GBM-BioDP overall architecture. The diagram represents a runtime view of the architecture of GBM-BioDP. The lower tier represents the sources of experimental and meta data, and external tools that are invoked to visualize the data. The middle tier represents how the data are processed, stored, and made available to the user. The right hand side of the middle tier represents the visualization “services” that are available at runtime to the user. These services are made available as web-services and are hosted on an Apache server. The higher tier represents the user interface, and is organized in a tabbed interface.

The visualizer was constructed as a tiered application. The high-level architecture is shown in Figure 1 and consists of three tiers. The lower tier represents the sources of experimental and meta data (as described in the previous section), and R (http://cran.r-project.org) [23] external tools that are invoked to analyze and visualize the data. The middle tier represents how the data are processed, stored, and made available to the user. Processing is done in R, the data are stored in an MySQL database, and the visualization options or “services” are made available to the user at runtime as web-services hosted on an Apache server. The higher tier represents the user interface, and is organized in a tabbed interface, and implemented in PhP and Java Script.

### Statistical tests

Statistical analyses were performed in R and the results are incorporated into visual displays. Four types of analyses are made: descriptive, prognostic, correlation, and predictive. These analyses are performed on the patients stratified by molecular subtypes as detailed earlier, as well as on the total cohort selected. Examples of these analyses are described in the online tutorial provided in GBM-BioDP website.

1. Descriptive analyses: Box and whiskers plots permit to graphically represent descriptive statistics of a continuous variable (e.g. gene). Here, box and whiskers plots are presented to visually compare distributions of a gene among the different molecular subtypes. When there is more than one molecular subtype, Welch’s test is used to evaluate the difference of gene’s expression among the subtypes.
2. Prognostic analyses: Prognostic analyses were performed at the targeted single molecule (gene, protein or miRNA) level or multiple selection criteria.
  A. Targeted prognostic analyses for chosen criteria include several statistical tests. These tests were conducted on GBM subtypes and total cohort with data previously converted to a common scale with a suitable z-score normalization. The prognostic impact of each expression measure is evaluated by means of univariate Cox proportional hazards model. Results are displayed according to the molecular subtype, and total cohort. Kaplan-Meier curves were then performed with expression values dichotomized according to the median value. Cox results corresponding to dichotomized values were displayed on the curve. An option is provided to group patients based on quartile selection of the expression measure.
  B. Multiple selection criteria: For a list of expression measures, two types of analyses were performed.
    - Cluster based prognostic analyses, in which patients were stratified by hierarchical clustering based on the selected list of molecules. Using silhouette width index, optimum clusters were selected. A log rank statistic was performed using Kaplan Meier curves to test the differences between the cluster groups. These analyses were performed using “NbClust” package in R.
    - Prognostic index estimation. The prognostic index (PI), also known as the risk score, is commonly used to generate risk groups. The PI is known as the linear component of the Cox model, PI = *β_1_x_1_+β_2_x_2_+…+β_p_x_p_* where *x_i_* is the expression value and the *β_I_* can be obtained from the Cox fitting. Each *β_I_* can be interpreted as a risk coefficient. The fitting is performed in R using the “survival” package. The risk groups were dichotomized by ordered PI by median value (higher values for higher risk) leaving equal number of samples in each group, or by quartile selection. For the resulting two groups, a log-rank test is performed.
3. Correlation analyses: A pairwise Pearson correlation analysis was performed for the user selected list of molecules. The results were displayed as heat map using hierarchical cluster analysis using average linkage distance metric.
4. Predictive analyses: To classify new patients or samples into the known molecular subtypes of GBM, two prediction methods were implemented. These analyses were performed using the Bioconductor packages “pamr” and “randomForest”.
  A. Prediction analysis of microarrays: Briefly, PAM [21] is performed using the nearest shrunken centroid method to classify GBM samples for the subset of genes submitted by the user. Classification error rates were estimated using k-fold cross validation method. Using the classification rule built by the training set, new samples were assigned to one of the subtypes. The results were displayed as prediction probabilities and class centroid scores for each gene.
  B. Random Forest: The Random Forest [22] is a machine learning method that generalizes the classification and regression tree method. Model validation was performed using “out of bag” estimation with bootstrapping, and new samples were predicted by the classification rule built by the model.

## Core Features

The interface is organized into modules. Currently the three main modules are centered on the concept of Genes, miRNAs, and (prediction) Models.

### Genes and miRNAs modules

Genes and miRNAs modules provide analogous features centered on the molecule (gene, miRNA) or molecule product (protein) in question.

**Figure 2:**
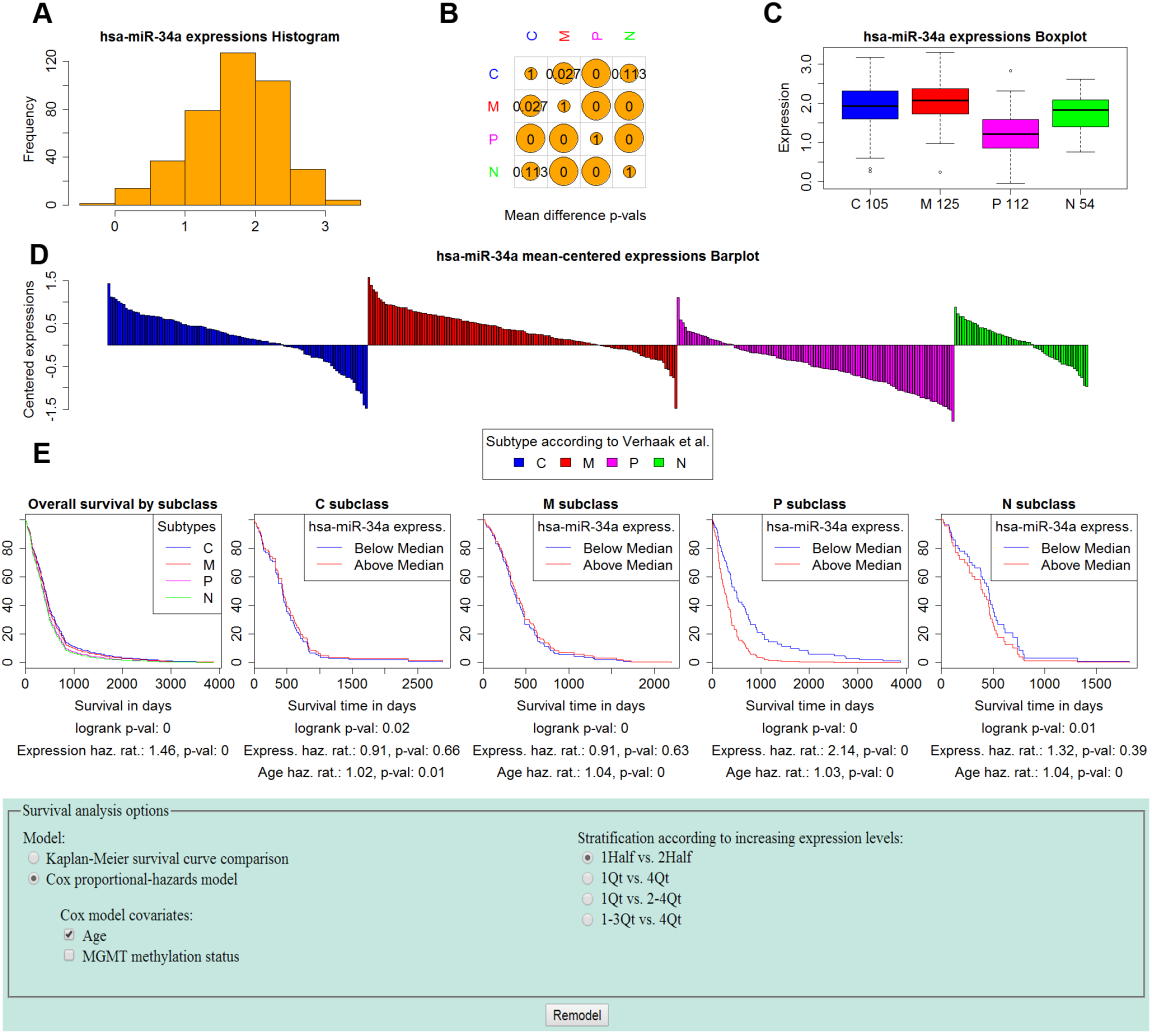
miRNAs module use case – mir-34a profile and survival analysis by GBM subtype stratification. mir-34a was recently identified (by Genovese et al. [26]) as a regulator of TGF-Beta in GBM, and as having prognostic value. Panels A–D show the distribution of mir-34a expression levels. Panel A shows the distribution over all GBM samples. Panel B tabulates the p-values of two-sided t-tests comparing the expression levels between subtypes. Panel C shows a boxplot of the mir-34a expression distribution for each subtype. The proneural subtype shows significantly lower expression compared to the other subtypes (the proneural p-values from Panel B are both zero). Panel D shows barplots of the expression for each group, centered around the entire GBM sample mean. Panel E shows the survival analysis results and options used. We perform a Cox proportional hazards survival analysis with mir-34a expression levels stratified as low if they are below the median and high otherwise, and Age as covariate. The analysis confirms the results of Genovese et al. who found lower expression of mir-34a in proneural patients to be associated with better prognosis. Indeed, the analysis shows that high levels of mir-34a expression are associated with a hazard ratio of 2.14 (p-value = 0, and logrank = 0).

**Figure 3:**
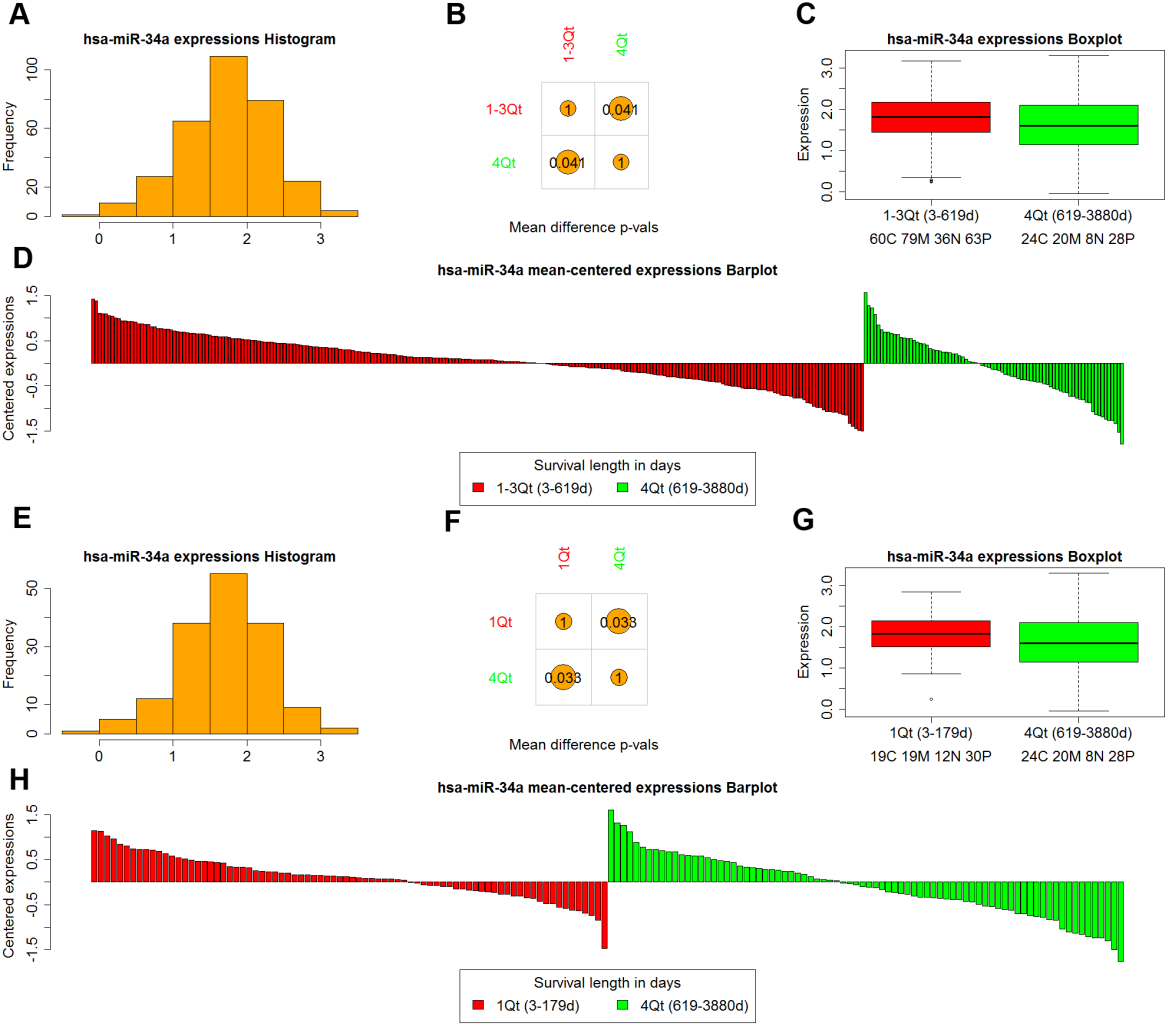
miRNAs module use case – mir-34a profile by survivorship stratification. We compare the expression levels of mir-34a in samples stratified by length of survival. Panels A–D shows comparison of 1–3 Quartiles (short survivors) versus 4th Quartile (long survivors). Panels E–H shows comparison of 1st Quartile (short survivors) versus 4th Quartile (long survivors). A, E show the histogram of expression distribution for all GBM patients. B, F show the p-values of the t-tests comparing expression of mir-34a in short and long survivors. C, G show the boxplots of expression for short and long survivors, and D, H show barplots of expressions mean-centered around the mean of the two groups. In both stratifications, long survivors (4th Quartile patients) express significantly lower levels of mir-34a compared to short survivors (p-val 0.041 when compared to 1–3 Quartile patients, and p-val 0.033 when compared to 1st Quartile patients).

1. Expression profiles can be visualized for single or multiple molecules. For single molecules, the expression profile visualization includes a histogram of the distribution of the zscore-expression values (Figure 2A), as well as comparisons of expressions among groups (Figure 2B, C, D). It is possible to compare expression among samples of different types (C, M, P, N), and therefore determine if a particular gene or miRNA has a role in dysregulation in one or more of the subtypes. Another alternative is to explore the expression of the molecule in groups of patients with different length of survival, therefore inspecting if the expression levels are markers of clinical outcome (Figure 3).

Expression profiles of multiple molecules can be inspected by constructing heatmaps that cluster according to similarity.

1. In conjunction with expression profiles, the user can perform survival analyses that consider the impact of a molecule’s expression levels on the length patient survival. When patients are stratified according to their molecular subtype (C, M, P, N) survival analysis can further stratify samples according to the expression levels of the molecule in question. Kaplan-Meier curves, or Cox proportional hazards models can be constructed that compare the impact of high or low expression values on the length of survival (Figure 2E). For Cox models, other covariates of regression can be considered in conjunction with the expression level classification. Age, and MGMT promoter methylation status have been shown to have prognostic value [24], and therefore options to include them in the model are available.

For multiple molecules, two types of survival analysis can be performed. First, the samples are clustered by similarity of expression profiles of the molecules in question, and Kaplan Meier analysis displays any differences in survival based on cluster membership. Second, a PI is generated for the user defined selection (genes, proteins or miRNAs). A Cox model is constructed with this prognostic index as covariate, and optional covariates for Age and MGMT promoter methylation status. The effect of different levels of the prognostic index is displayed for the full cohort, and for each GBM subclass.

**Figure 4:**
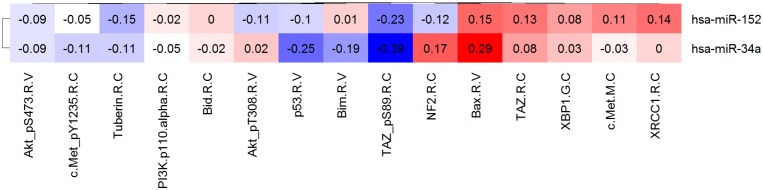
Use case from miRNAs module – miRNA targets. Correlation of mir-34a predicted targets, and p53. Color red represent positive correlation, whereas blue color represent negative correlation. The cells are annotated with the correlation values. p53 and mir-34a expression are anti-correlated, which indicates a possible suppressor role of mir-34a.

1. Regulator-target relationships among miRNA and gene products can be explored by constructing expression correlation heatmaps between miRNAs and gene products that are computationally predicted to be in such a relationship based on sequence similarity [25]. Since sequence similarity alone generates spurious results given the short length (6–8 nucleotide) of miRNAs seed regions, more evidence of direct “sponge” interactions can be determined by considering the correlation of the expression levels. In a typical direct interaction the expression levels of the two move in opposite directions, where higher levels of miRNA expression result in lower levels of mRNA and ultimately protein expression, and lower levels of miRNA result in higher levels of mRNA and protein expression (Figure 4).

### Models

Models module enables classification of user supplied gene or miRNA expression data into the four molecular subtypes of GBM by supervised classification methods. Currently we provide options for two classification methods (PAM and Random Forest). The results include predicted classification and associated probabilities, plots for visualizing the profiles of the top classifiers, and heatmaps of correlation between model and user input data.

#### 1. Biological Validation – mir-34a and its Prognostic Value

Here we give an example of biological validation from miRNAs module, demonstrating how the profiles of genes and miRNAs recently identified in the literature as having an important mechanistic and prognostic role in GBM can be inspected using GBM-BioDP.

Micro RNAs have a key regulatory role, directly and indirectly impacting the expression level of genes. In particular, mir-34a has been recently studied in connection with its regulatory role in GBM. Genovese et al. [26], reported genes and molecules markedly dysregulated amongst the GBM subtypes, and identified mir-34a as one of the key regulators in GBM. mir-34a is overall overexpressed in GBM; however, amongst samples with proneural profile, mir-34a was found to have lower levels of expression. More interestingly, lower levels of expression of mir-34a in the proneural subtype were associated with better prognosis.

Figure 2 shows the GBM-BioDP profile of mir-34a. Consistent with Genovese et al.’s [26] findings, mir-34a is overexpressed across GBM samples – zscores of the majority of the samples are above 1.5 (Figure 2A, C). Moreover, proneural samples show significantly lower expression of mir-34a compared to the other types of GBM (pairwise t-test p-values for proneural versus other subtypes are 0, Figure 2B, 2C). As seen from the survival analysis (Figure 2E), stratification of samples along low and high expression of mir-34a show significant differences in survival length for the proneural type. This is true even when considering the effects of the age covariate in the COX model on prognosis supporting that younger age at diagnosis is associated with better prognosis. A higher mir-34a is associated with a hazard ratio of 2.14 (pvalue = 0, and logrank = 0).

A more recent study by Sasaki et al. [27]shed more light on mir-34a’s involvement in GBM. Sasaki et al. studied the effects of radiation on GBM cells, and reported that lower doses of radiation (30 Gy) cause the levels of mir-34a to decrease compared to controls, whereas higher doses of radiation (60 Gy) increased (18.7 times) the levels of mir-34a. The levels of mir-34a were inversely correlated with the levels of p53. They hypothesize that mir-34a directly regulates p53, with the low mir-34a expression levels under 30 Gy irradiation having the effect of increased p53 levels, and therefore increased apoptosis induction; the reverse is thought to be the case for higher doses of irradiation.

In light of Sasaki’s study, we inspected whether lower levels of mir-34a are in general associated with better survival. Figure 3 shows a comparison of the expression levels between patients stratified according to the length of survival. The patients with longest survival (4th quartile) exhibit significantly lower levels of mir-34a (t-test p-value 0.041 for comparison of 4th quartile to the 1–3 quartiles, and 0.033 for comparison of 4th quartile versus 1st quartile, Figure 3B, F). Moreover, the prognostic significance of lower levels of mir-34a for proneural patients is likely tied to their p53 mutation status which is frequently mutated in proneural GBM, whereby in patients with mutated p53, the effect of increased levels of mir-34a are more detrimental than in patients with wild type p53.

To inspect the relationship between mir-34a, and p53 protein expression levels we constructed an expression correlation heatmap between mir-34a, and its predicted targets in Microcosm. Note that p53 is not one of the top predicted targets of mir-34a (based on sequence similarity), therefore we included in the heatmap mir-152, which is one of the strongest predicted regulators of p53 (see predicted regulators of p53 from the Genes module). The correlation heatmap (Figure 4) points to an inverse relationship between mir-34a and p53 expression levels.

#### 2. Hypothesis generation – Molecular classification of GBM clinical data

GBM-BioDP can be used to predict the molecular subtypes of new experimental, or clinical samples into known molecular subtypes, which can aid in improving understanding of GBM neurogenesis, constructing experimental models, and tailoring therapies. We illustrate the use of the tool by looking at the classification of grade IV gliomas (GSE4271) that were used by Phillips et al. [28] to construct their high grade glioma neurogenesis model.

**Figure 5:**
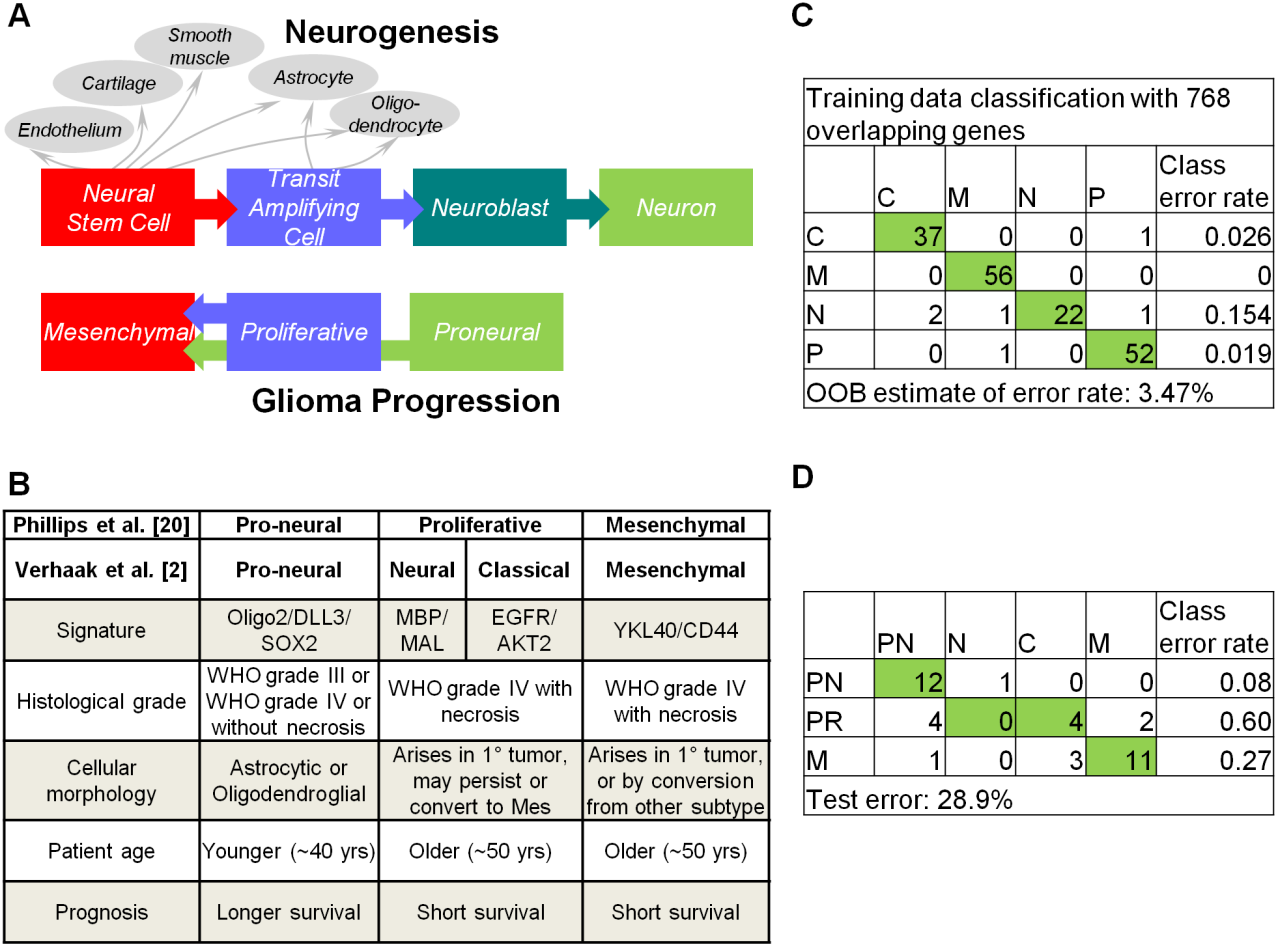
Hypothesis generation – Molecular classification of GBM clinical data. A. Model depicting parallels between tumor subtypes and stages in neurogenesis [28]. B. Main features of tumor subtypes [6], [28]. C. Random forest model “out of bag” error rates for training data using 201 samples from [6] and the corresponding 768 gene expression measurements common between the training and validated data. D. Summary of error rates for the predicted subtypes of validated data. Abbreviations RF: random forest, C: classical, M: mesenchymal, N: neural, P: proneural.

Phillips et al. [28] proposed a neurogenesis model of high grade gliomas in which gliomas arise from a cell type with neural stem cell properties (Figure 5A). They documented three prognostic subclasses of glioma (*pronerual*, *proliferative*, and *mesenchymal*), which were associated with key stages in neurogenesis and implicated signaling pathways critical in regulation of forebrain neurogenesis and tumor aggressiveness. The authors also demonstrated a frequent pattern of glioma progression into the mesenchymal phenotype, a state associated with robust angiogenesis. More recently, Verhaak et al. [6] expanded on Phillips et al.’s classification by associating specific gene alterations with each subclass and identifying two additional subtypes (splitting the proliferative class into *neural* and *classical*; see Figure 5B). The recognition that GBM consists of subtypes varying in molecular circuitry and biological behavior suggests that no therapy can be universally efficacious and that meaningful therapeutic gain can only be attained by customizing the therapy to the underlying molecular circuit.

We tested the molecular class prediction based on the expression profiles of an independent set of patients with grade IV gliomas (GSE4271). The data included patients from primary or recurrent GBM presenting with or without necrosis (see Figure 5B for a summary of clinical characteristics). The microarray data were pre-processed and filtered to contain features common to the Verhaak et al. training data and highly variable and representative samples for each subtype (the samples were filtered by the feature reduction method of principal component analysis). This resulted in 38 samples and 768 features that served as test set. We computed the classification prediction model using the random forest option, which resulted in an overall training data error rate of 3.47% (Figure 5C). We recalculated prediction error rates from Phillips et al [28] data using their test set. The results are summarized in Figure 5D.

Our model correctly predicted proneural subtypes with an error rate of 0.08. The median age of patients in this group is 31 years, supporting earlier findings that patients classified as proneural are of younger age group. The majority of mesenchymal samples were also predicted into the correct class (11/14, error rate of 0.27). However, wide heterogeneity was observed for the proliferative class. Recall that the proliferative class designated by Phillips et al. was further subclassified into neural and classical by Verhaak et al. (Figure 5B). In our analysis, out of 10 samples, 4 were classified as classical, 4 proneural and 2 mesenchymal. The samples predicted to be proneural had age range of 30–32 years, suggesting that age may have a dominant effect on gene expression patterns. On examination, the two samples that were predicted as mesenchymal are primary tumors with necrosis. Clinical studies indicate that of all clinical, neuroimaging, and histopathological characteristics (including age), necrosis that is visible on magnetic resonance imaging scans has the greatest prognostic value and is inversely related to patient survival [29], [30]. Therefore it is likely that these patients were predicted to have mesenchymal type due to having molecular features of aggressive phenotype. These findings shed new light on the original classification of Phillips et al. [28] and illustrate the potential of the tool for predicting prognostic clinical subtypes and constructing clinically relevant models.

## Summary and Future Work

Advances in biotechnology have brought about accumulation of large scale cancer genomic data, in turn introducing the challenge of making the data available to a wide array of researches, scientists, and clinicians. While several impressive resources that offer visualization options for various omic data exist, no such tool can be used in all possible scenarios. We present a new resource for visualizing GBM TCGA data, which aims at providing streamlined visualizations of common scenarios when investigating molecular profiles of glioblastoma, and how these profiles are associated with clinical outcomes.

The resource offers integrated views of several dimensions of TCGA data, such as mRNA, protein, and miRNA expression, exploration of target-regulator relationships, as well as provides predictive models for user input data. Molecular profiles can be explored based on a number of sample stratification options (from GBM subtypes to different clinical outcomes). Conversely, clinical outcomes can be explored for samples exhibiting different molecular profiles. The resource can be used for biological validation, as well as generation of new hypothesis.

We are currently developing options for integrating views from other TCGA omic data including copy number, SNP, and DNA methylation. Another active area of development is integration at the functional and biological pathway enrichment level.
